# Prevalence and correlates of job and insurance problems among young breast cancer survivors within 18 months of diagnosis

**DOI:** 10.1186/s12885-020-06846-w

**Published:** 2020-05-18

**Authors:** Michelle J. Naughton, Chloe M. Beverly Hery, Sarah A. Janse, Elizabeth Z. Naftalis, Electra D. Paskett, Kimberly J. Van Zee

**Affiliations:** 1 Division of Cancer Prevention and Control, Department of Internal Medicine, College of Medicine, The Ohio State University, Columbus, OH 43210 USA; 2 Division of Epidemiology, College of Public Health, The Ohio State University, Columbus, OH 43210 USA; 3 Center for Biostatistics, Department of Biomedical Informatics, The Ohio State University, Columbus, OH 43210 USA; 4 Health Texas Community Health Services Corporate Director of Breast Services, Dallas, TX 75001 USA; 5 Breast Service, Department of Surgery, Memorial Sloan Kettering Cancer Center, New York, NY 10065 USA

**Keywords:** Breast cancer, Employment, Insurance, Young survivors

## Abstract

**Background:**

The prevalence and correlates of job and insurance problems were examined among a cohort of young U.S. breast cancer survivors during the first 18-months following diagnosis.

**Methods:**

Participants were 708 women diagnosed at ≤45 years with stage I-III breast cancer. 90% were non-Hispanic white, 76% were married/partnered and 67% had ≥4-year college degree. Univariable and multivariable logistic regression examined the associations between demographic, lifestyle and clinical factors with job and insurance problems.

**Results:**

18-months after diagnosis, 56% of participants worked full-time, 16% part-time, 18% were homemakers and/or students, 4.5% were unemployed, and 2.4% were disabled. The majority (86%) had private insurance. Job-related problems were reported by 40% of women, and included believing they could not change jobs for fear of losing health insurance (35.0%), being fired (2.3%), and being demoted, denied promotion or denied wage increases (7.8%). Greater job-related problems were associated with being overweight vs. under/normal weight (*p* = 0.006), income <$50,000/per year (*p* = 0.01), and working full-time vs. part-time (*p* = 0.003). Insurance problems were reported by 27% of women, and included being denied health insurance (2.6%), health insurance increases (4.3%), being denied health benefit payments (14.8%) or denied life insurance (11.4%). Insurance problems were associated with being under/normal weight vs. obese (*p* = 0.01), not being on hormone therapy (*p* < 0.001), and a tumor size > 5 cm vs. < 2 cm (*p* = 0.01).

**Conclusions:**

Young survivors experienced significant job- and insurance-related issues following diagnosis. To the extent possible, work and insurance concerns should be addressed prior to treatment to inform work expectations and avoid unnecessary insurance difficulties.

## Background

The diagnosis and treatment of breast cancer can result in major changes in a woman’s daily routine, and for many younger women, this includes their employment status [1]. Career development and individual and family transitions during early and mid-adulthood may be interrupted by diagnosis and treatment, which may lead to long-term negative impacts [2]. Despite intentions to continue working while receiving treatment, a majority of women have to reduce their paid work hours at least temporarily, particularly within 6 months of diagnosis [1]. Although many cancer survivors are able to return to work fully after treatment, some are at risk of employment and insurance discrimination [3]. Concerns among cancer survivors include a reduced ability to change jobs or get promoted, denied or reduced health insurance coverage, and changing health care providers [3]. Even among insured populations, continuing coverage frequently influences employment decisions [4], and younger patients have been shown to be at a higher risk for financial difficulties [4–6].

Several studies have examined employment issues following cancer diagnosis and treatment. A systematic review indicated that survivors had a significantly higher risk of unemployment and early retirement, and were less likely to be re-hired [7]. Work environment, type of work performed, and increased fatigue post-treatment contributed to reasons why women were less likely to return to work after breast cancer treatment [8–10]. Physician advice about flexible working conditions, and meeting with employers to discuss work requirements during and/or after treatment, have been associated with a greater likelihood of women being employed [11]. However, women who do return to work post-treatment may report fewer opportunities for promotion [12, 13], reduced job satisfaction [13] and discrimination [14].

Factors associated with return to work after treatment include perceived employer accommodations for treatment and recovery, younger age at diagnosis, higher education, fewer physical symptoms, and lack of surgical treatment [7, 15, 16]. Similarly, general employment after breast cancer is associated with a higher educational level, higher household income, fewer comorbidities, earlier disease stage and less extensive surgery [9, 10, 12, 17–20]. Type of treatment is also associated with work status in that women who receive chemotherapy and have positive nodes are less likely to be employed in comparison to women who receive adjuvant endocrine therapy [8].

The availability of health insurance can be a significant predictor of women’s return to work following breast cancer treatment. Unlike other countries, the United States (U.S.) does not have national health insurance, except for persons aged 65 or older (Medicare) or support for vulnerable populations, such as those with disabilities or very low incomes (Medicaid). Health insurance in the U.S. is closely tied to employment, with individual employers offering group insurance options to their employees to purchase. Employed persons without employer-sponsored health care must buy insurance coverage from private health insurers, whose products can differ in what types of illnesses and services are covered in the plans, often with varying premiums and co-pays that individuals have to pay.

With a high proportion of young women being employed, issues related to work and health insurance during and after cancer diagnosis and treatment are highly relevant. Women without employer sponsored health insurance have been found to leave work at a significantly higher rate compared to those with employer-sponsored health insurance [21]. Women with breast cancer who have health insurance through their spouse are also significantly more likely to quit work or reduce their weekly work hours at multiple time points post-diagnosis compared with women who have insurance through their employer [22].

Many women report that dealing with employment and insurance issues is extremely stressful, and has adverse effects on their lives [23]. This paper focuses on the experience of a cohort of young breast cancer survivors, during the first 18 months post-diagnosis.

## Methods

This study is a secondary analysis of data from the Menstrual Cycle Maintenance and Quality of Life After Breast Cancer Treatment Study, a multi-center, longitudinal observational study [24]. Participant inclusion criteria were female patients between the ages of 18 and 45 years who had been diagnosed with stage I-III invasive breast cancer within the previous 8 months. Patients were excluded if they had any prior or concurrent history of any cancer, excluding basal or squamous cell skin carcinoma and stage 0 cervical cancer. Participants were required to have regular menstrual cycles at the time of diagnosis in order to examine primary aims regarding menstrual cycling, fertility, and menopause post-breast cancer treatment. Thus, women who had a previous hysterectomy were ineligible for this protocol.

Recruitment to this study began in January 1998 and ended in November 2005. Participants (*N* = 836) were enrolled through five sites located at the Memorial Sloan-Kettering Cancer Center in New York City, New York; M.D. Anderson Cancer Center in Houston, Texas; University of Texas –Southwestern, Dallas, Texas; Presbyterian Hospital in Dallas, Texas; and the Wake Forest University Baptist Medical Center in Winston-Salem, North Carolina. Patients from each clinical center were identified soon after diagnosis using tumor or surgical registries and patient or physician referrals. Baseline forms were completed at the time of study recruitment at the participating sites. All follow-up data collection was conducted via mail through the study coordinating center at the Wake Forest University School of Medicine. Written informed consent was obtained from all individual participants included in the study. This study was approved by the Institutional Review Board of each participating site as well as the U.S. Army Medical Research and Materiel Command Human Subjects’ Committee.

### Measures

#### Demographics

Information was collected during study enrollment on age, marital status, race/ethnicity, educational background, income, employment status, and insurance status.

#### Employment concerns

Participants reported their employment status at study entry and at approximately 18 months post-diagnosis. Participants reported whether they had been laid off, demoted, denied a promotion or denied a wage increase, had their work responsibilities limited unnecessarily, lost health insurance due to sick leave, and whether they believed any of these concerns was related to their cancer diagnosis. In addition, the participants were asked if they believed they could not change jobs for fear of losing their health insurance. Participants could report more than one concern.

The job and insurance-related questions came from a questionnaire developed through the Cancer and Leukemia Group B (CALGB), one of the larger U.S. cooperative groups funded by the National Cancer Institute (NCI). This group was merged into the Alliance for Clinical Trials in Oncology in 2010, after the reorganization of the NCI’s community oncology research program (ncorp.cancer.gov). The questionnaire items were developed from existing publications and other scales of job and insurance issues after cancer/illness, and were pretested and validated with cancer survivors in the CALGB.

#### Insurance concerns

At approximately 18 months post-diagnosis, participants reported whether they had experienced any of the following since their cancer diagnosis and treatment: denial of health or life insurance, increases in health or life insurance rates, denial of a health benefit payment, or difficulty changing from group health to an individual health plan, if applicable. Participants could report more than one area of concern.

#### Medical chart review

Extensive chart reviews were performed at the recruiting institution. Information was obtained on the patients’ height and weight, smoking status, date and technique of breast cancer diagnosis, tumor size, location, grade, estrogen receptor (ER) status (positive or negative), progesterone receptor (PR) status (positive or negative), number of nodes examined, number of positive lymph nodes, type of definitive cancer surgery, and reconstructive surgery, if any. Any further surgery, such as delayed reconstruction or oophorectomy, was likewise obtained. Chemotherapy information (dates, drugs, and dosages in milligrams [mg]) was gathered from medical oncology office records. For those receiving radiation, dose per treatment, treatment area (breast, boost site, axilla, chest wall, and/or supraclavicular), total dosage and duration of treatment, were recorded. Endocrine therapies, such as tamoxifen and leuprolide, were recorded with dates, routes of administration, and dosages.

### Statistical methods

Baseline demographic and clinical characteristics were calculated using counts and percentages for categorical variables. Frequencies of the participants’ responses to the job and insurance questions were also tabulated. Univariable and multivariable analyses were performed using logistic regression to examine the association between the demographic, lifestyle, and clinical characteristics of the participants, and job and insurance problems. Univariable analyses were performed as a first step. Variables included in the univariable analyses for “any job problem” and “any insurance problem” included: demographics (age, race, education, employment status, health insurance, income, marital status); lifestyle factors (body mass index [BMI]; smoking status); cancer treatment (chemotherapy [yes/no], radiation [yes/no], hormonal therapy [yes/no]); and cancer severity (estrogen/progesterone status, histologic grade, number of positive lymph nodes; cancer stage; tumor size). Variables (or subcategories of variables) that obtained a conservative *p*-value of <.30 were retained in the multivariable analyses. Thus, the univariable analyses resulted in eliminating, chemotherapy, radiation and the number of positive lymph nodes from any further analyses of either employment or insurance concerns. Correlations among predictors were also explored. To avoid issues with multicollinearity, estrogen/progesterone status was omitted from the multivariable models due to high correlation with hormonal therapy. We also found high correlations between stage and the number of positive lymph nodes and tumor size, but only one of each of these variables was included in each of the multivariable analyses based on preliminary univariable results.

Multivariable logistic regression was performed next, and both full and reduced models were completed. For the multivariable logistic regression analysis examining “any job problem,” age, marital status, race, BMI, education, income, employment status, insurance type, current smoking status, hormonal therapy, tumor size, and histologic grade were included in the model. For the logistic regression examining “any insurance problem,” age, marital status, race, BMI, education, income, employment status, insurance type, current smoking status, cancer stage, hormonal therapy, and tumor size, were included in the model. Thus, the models for job problems and insurance problems varied based on the results of the univariable analyses for each problem type. Full models including all variables were completed first, and then stepwise selection was used to calculate reduced, final multivariable models for the insurance and job-related problems. All analyses were conducted using SAS 9.4 (SAS Institute Inc., Cary, North Carolina, USA). Statistical significance levels were set at a *p*-value of < 0.05.

## Results

A total of 708 women (85% of the original sample) completed the employment and insurance questions and were included in these secondary analyses. The majority were married or partnered, and 54% were < 40 years old at diagnosis (Table 1). The participants were well-educated with 67% having obtained at least a 4 year college degree, including 21% with a master’s degree and 8% with a doctoral degree. Ninety percent of the participants identified themselves as non-Hispanic white, with 4% identifying as African-American, 4% Hispanic, and 2% Asian Pacific Islander. The majority of participants were diagnosed with breast cancer at stage I or II, with 55% having no lymph node involvement. Approximately half of the women had a mastectomy. Most (87.7%) received chemotherapy.

**Table 1:** Patient Demographic and Clinical Characteristics (*N* = 708)

Variable^**a**^	Total (***n*** = 708)
Marital Status	
Never married	106 (15%)
Presently married	503 (71%)
Marriage-like relationship	38 (5.4%)
Divorced	49 (6.9%)
Separated	8 (1.1%)
Widowed	4 (0.6%)
Education	
< High School Graduation	6 (0.8%)
High school diploma or GED	42 (5.9%)
Some college/Associate degree	183 (25.8%)
College graduate (B.A. or B.S.)	208 (29.4%)
Some graduate or professional school	61 (8.6%)
Master’s degree	149 (21%)
Doctoral degree	58 (8.2%)
Race	
Non-Hispanic White	636 (89.8%)
Black/African American	27 (3.8%)
Hispanic	27 (3.8%)
Asian/Pacific Islander	18 (2.5%)
Income	
< $20,000	43 (6.1%)
$20,000 - $34,999	44 (6.2%)
$35,000 - $49,999	76 (10.7%)
$50,000 - $74,999	133 (18.8%)
$75,000 - $99,999	131 (18.5%)
≥ $100,000	267 (37.7%)
Preferred not to answer	14 (2%)
Employment status	
Full-time homemaker	117 (16.5%)
Employed full-time	398 (56.2%)
Employed part-time	112 (15.8%)
Disabled	17 (2.4%)
Unemployed/looking	32 (4.5%)
Retired	3 (0.4%)
Student	8 (1.1%)
Other	19 (2.7%)
Occupation (full- and part-time workers)	
Professional/Technical	289 (55.9%)
Manager, administrator, or proprietor	73 (14.1%)
Clerical	54 (10.4%)
Sales occupation	37 (7.2%)
Service occupation	12 (2.3%)
Skilled craft, service repair	2 (0.4%)
Laborer	5 (1.0%)
Military	3 (0.6%)
Equipment/vehicle operator	1 (0.2%)
Other	41 (7.9%)
Insurance Type^b^	
Health Maintenance Organization	200 (28.2%)
Group Healthcare Plan	410 (57.9%)
Veterans Affairs/ Military sponsored	8 (1.1%)
Individual/Private Health Insurance	31 (4.4%)
Medicaid	13 (1.8%)
Disability	8 (1.1%)
None	8 (1.1%)
Other	45 (6.4%)
Age at diagnosis	
< 30 years	44 (6.2%)
30–34.9 years	130 (18.4%)
35–39.9 years	208 (29.4%)
40–45 years	326 (46%)
Smoke Currently	
No	644 (91%)
Yes	63 (8.9%)
Body Mass Index (BMI)	
Underweight (< 18)	15 (2.1%)
Normal (18–24.9)	449 (63.4%)
Overweight (25–29.9)	135 (19.1%)
Obese (≥ 30)	87 (12.3%)
Missing	22 (3.1%)
Lumpectomy	
No	155 (21.9%)
Yes	548 (77.4%)
Mastectomy	
No	355 (50.1%)
Yes	351 (49.6%)
Radiation Therapy	
No	208 (29.4%)
Yes	498 (70.3%)
Chemotherapy	
No	87 (12.3%)
Yes	621 (87.7%)
Hormonal Therapy	
No	271 (38.3%)
Yes	435 (61.4%)
Node(s) Positive	
0	392 (55.4%)
1–3	205 (29%)
4–9	67 (9.5%)
10+	42 (5.9%)
Tumor Size	
< 2 cm	428 (60.5%)
2–5 cm	210 (29.7%)
> 5 cm	29 (4.1%)
Missing	41 (5.8%)
Stage of Cancer	
I	302 (42.7%)
II	365 (51.6%)
III	40 (5.6%)
Histologic Grade**:**	
I	51 (8.8%)
II	189 (32.5%)
III	341 (58.7%)
IV	1 (0.1%)
Estrogen/Progesterone Status:	
**−/−**	201 (29.3%)
−/+	23 (3.4%)
**+/−**	69 (10.1%)
**+/+**	393 (57.3%)

^a^Not all participants answered all questions

^b^More than one type of insurance could be marked

At 18 months post-diagnosis, 72% were employed either full (56%) or part-time (15.8%), 2.4% were disabled, and 4.5% were unemployed or looking for work. The majority of women (56%) were employed in professional or technical occupations, 14% were managers or administrators, 10.4% percent were in clerical occupations, and 7% were in sales. In terms of insurance, approximately 86% of the women were enrolled in a Health Maintenance Organization (HMO) or a group health insurance plan through either their own or a spouse’s/partner’s employer-based health care plan. Approximately 4% had individual health insurance, and 3% had Medicaid or disability insurance. Only 1% had no health insurance.

### Job/employment

Participants were asked if they experienced any job-related problems that they believed were related to their cancer diagnosis/treatment (Table 2). Overall, 39.7% (*n* = 205) of the women reported experiencing at least one job-related problem following their diagnosis and treatment. The problems reported included believing they could not change jobs for fear of losing health insurance (35.0%), being fired (2.3%), and 7.8% reported being demoted, denied promotion or denied wage increases. Having their work responsibilities limited unnecessarily was reported by 3.9% of the women.

**Table 2:** Employment and Insurance Related-Problems as a Result of Breast Cancer Diagnosis and Treatment

**Job Problems**^**a**^**Related to Cancer Diagnosis or Treatment (*N*** **= 516)**	N^**b**^	n	%
Any job related problem^c^	516	205	39.7
Believed they could not change jobs for fear of losing their health insurance	514	180	35.0
Lost health insurance	515	4	0.8
Fired or laid off from job	516	12	2.3
Demoted	513	5	1.0
Denied a promotion	514	13	2.5
Denied a wage increase	514	22	4.3
Had work responsibilities limited unnecessarily	514	20	3.9
**Insurance Problems Related to Cancer Diagnosis or Treatment (*N*** **= 708)**			
Any insurance related problem^c^	707	190	26.8
Denied health insurance	705	18	2.6
Health insurance rates increased	703	30	4.3
Had a health benefit payment denied	702	104	14.8
Had trouble changing from group health to individual health insurance	685	20	2.9
Life insurance rates increased	690	10	1.4
Denied life insurance	702	80	11.4

^a^Only includes participants who were employed for pay full- or part-time

^b^Not all participants answered all questions

^c^Participants could report more than one job-related or insurance-related problem

Demographic and clinical correlates of the participants’ job and employment concerns after diagnosis were examined using multivariable logistic regression. Both the full and reduced models are presented in Table 3. In the final reduced model, BMI, income level, and employment status were significantly associated with job problems. Overweight and obese women reported more job problem than those who were under/normal weight, yet only the overweight category reached statistical significance (OR: 2.09, 95% CI: 1.23–3.56, *p* = 0.006). An income of >$50,000 was consistently associated with lower odds of job-related problems compared to an income of <$50,000 (*p* values < 0.04). Additionally, the odds of job-related problems were about 58% less in women working part-time compared to those with full-time employment (OR: 0.42; 95% CI: 0.23–0.74, *p* = 0.003).

**Table 3:** Full and Reduced Stepwise Models of Multivariable Logistic Regression of Demographic and Clinical Factors Associated with any Job problem after Breast Cancer Diagnosis and Treatment

	Full Model		Reduced Model	
Participant Characteristics	OR (95% CI)	p-value	OR (95%CI)	*p*-value
Age		0.51	-----**	
< 30 years	0.96 (0.38, 2.43)	0.94		
30–34.9 years	1.58 (0.85, 2.94)	0.15		
35–39.9 years	1.18 (0.70, 2.00)	0.53		
40–45 years	reference			
Marital status		0.70	-----	
Divorced/Separated/Widowed	0.73 (0.30, 1.76)	0.48		
Married/Partnered	0.80 (0.43, 1.47)	0.47		
Never Married	reference			
Race		0.63	-----	
Non-Hispanic White	0.81 (0.34, 1.91)	0.63		
Other	reference			
Body Mass Index (BMI)		0.02		0.02
Under/Normal Weight (< 25)	reference		reference	
Overweight (25–29.9)	2.18 (1.24, 3.86)	0.007	2.09 (1.23, 3.56)	0.006
Obese (≥ 30)	1.47 (0.71, 3.03)	0.30	1.41 (0.72, 2.77)	0.32
Education		0.18	-----	
High School or less	reference			
Some College	0.59 (0.20, 1.73)	0.34		
College Degree	0.84 (0.29, 2.44)	0.75		
Post-Graduate	1.17 (0.41, 3.37)	0.77		
Income		0.06		0.01
< $50,000	reference		reference	
$50,000–$74,999	0.41 (0.20, 0.84)	0.01	0.39 (0.21, 0.74)	0.004
$75,000–$99,999	0.49 (0.24, 1.01)	0.05	0.52 (0.28, 0.98)	0.04
$100,00 or higher	0.44 (0.22, 0.88)	0.02	0.45 (0.26, 0.78)	0.005
Employment Status		0.008		0.003
Full-time	reference		reference	
Part-time	0.44 (0.24, 0.81)	0.008	0.42 (0.23, 0.74)	0.003
Insurance Type		0.27	-----	
Group Heath	reference			
HMO	0.57 (0.33, 0.97)	0.04		
Other	0.74 (0.36, 1.51)	0.41		
More than 1 insurance type	1.61 (0.34, 7.53)	0.55		
None	N/A^a^			
Current Smoker		0.35	-----	
No	reference			
Yes	1.50 (0.64, 3.52)	0.35		
Hormonal Therapy		0.62	-----	
No	reference			
Yes	0.89 (0.56, 1.42)	0.62		
Tumor Size		0.35	-----	
< 2 cm	reference			
2–5 cm	1.41 (0.88, 2.27)	0.16		
> 5 cm	1.33 (0.37, 4.72)	0.66		
Histologic Grade		0.47	-----	
I	reference			
II	0.60 (0.26, 1.38)	0.23		
III	0.86 (0.38, 1.93)	0.72		
IV	N/A			

^a^Variable levels without enough counts in cells were not included and are referenced with “N/A”

**Variables not included in the reduced model are referenced with dashes

### Insurance

Overall, 26.8% (*n* = 190) of the women experienced at least one insurance-related problem (Table 2). Health insurance was denied for 2.6% (*n* = 18), and 4.3% (*n* = 30) reported health insurance rate increases after their diagnosis. Health benefit payments for cancer treatment were denied for 14.8% (*n* = 104). Approximately 3% (*n* = 20) reported that they had trouble changing from group health to individual health insurance, and 11.4% (*n* = 80) reported being denied life insurance.

Similar to employment concerns, multivariable logistic regression was used to examine factors associated with the participants’ insurance concerns following diagnosis/treatment. Both full and reduced multivariable logistic regression models were run (Table 4). In our final reduced model, BMI, hormone therapy, and tumor size were significantly related to insurance-related problems. Being obese versus of normal weight was associated with lower odds of insurance problems (OR: 0.43, 95% CI: 0.22–0.83, *p* = 0.01), as was the use of adjuvant hormone therapy (OR: 0.48, 95% CI: 0.34–0.69, *p* < 0.0001). A tumor size >5 cm was associated with about 3.1 times the odds of insurance-related problems compared to women with tumors < 2 cm (OR: 3.11, 95% CI: 1.30–7.42, *p* = 0.01).

**Table 4:** Full and Reduced Stepwise Models of Multivariable Logistic Regression of Demographic and Clinical Factors Associated with any Insurance Problem after Breast Cancer Diagnosis and Treatment

	Full Model		Reduced Model^a^	
Participant Characteristics	OR (95% CI)	*p*-value	OR (95%CI)	*p*-value
Age		0.70	-----	
< 30 years	0.94 (0.42, 2.12)	0.89		
30–34.9 years	1.31 (0.79, 2.17)	0.30		
35–39.9 years	0.99 (0.63, 1.55)	0.95		
40–45 years	reference			
Marital status		0.99	-----	
Divorced/Separated/Widowed	1.00 (0.45, 2.20)	0.99		
Married/Partnered	0.98 (0.55, 1.75)	0.94		
Never Married	reference			
Race		0.87	-----	
Non-Hispanic White	1.06 (0.54, 2.06)	0.87		
Other	reference			
Body Mass Index (BMI)		0.05		0.04
Under/Normal Weight (< 25)	reference		reference	
Overweight (25–29.9)	0.86 (0.53, 1.40)	0.53	0.84 (0.53, 1.33)	0.46
Obese (≥ 30)	0.42 (0.21, 0.83)	0.01	0.43 (0.22, 0.83)	0.01
Education		0.34	-----	
High School or less	reference			
Some College	1.93 (0.67, 5.53)	0.22		
College Degree	2.50 (0.87, 7.17)	0.09		
Post-Graduate	2.29 (0.79, 6.65)	0.13		
Income		0.31	-----	
< $50,000	reference			
$50,000–$74,999	1.16 (0.63, 2.14)	0.63		
$75,000–$99,999	0.65 (0.33, 1.27)	0.21		
$100,00 or higher	0.99 (0.54, 1.81)	0.97		
Employment Status		0.22	-----	
Full-time	reference			
Homemaker^a^	0.78 (0.44, 1.40)	0.41		
Part-time	1.17 (0.69, 1.99)	0.56		
Other	1.73 (0.91, 3.30)	0.09		
Insurance Type		0.84	-----	
Group Heath	reference			
HMO	0.77 (0.49, 1.20)	0.25		
Other	0.97 (0.54, 1.74)	0.91		
More than 1 insurance type	0.85 (0.16, 4.53)	0.85		
None	1.08 (0.16, 7.17)	0.93		
Current Smoker		0.91	-----	
No	reference			
Yes	0.96 (0.46, 2.00)	0.91		
Cancer Stage		0.41	-----	
I	reference			
II	0.73 (0.44, 1.22)	0.23		
III	1.04 (0.36, 3.00)	0.94		
Hormonal Therapy		0.0004		< 0.0001
No	reference		reference	
Yes	0.50 (0.34, 0.74)	0.0004	0.48 (0.34, 0.69)	< 0.0001
Tumor Size		0.10		0.02
< 2 cm	reference		reference	
2–5 cm	1.63 (0.96, 2.75)	0.07	1.33 (0.89, 1.97)	0.16
> 5 cm	2.69 (0.82, 8.85)	0.10	3.11 (1.30, 7.42)	0.01

^a^Variables not included in the reduced model are referenced with dashes

## Discussion

This cohort of young breast cancer survivors reported significant job- and insurance-related problems following their diagnosis. In general, these participants were highly educated with 67% having at least a 4-year college degree, and 86% having health insurance through their own or their spouse’s/partner’s employer. Approximately 70% of these women were employed in professional or administrative positions, which might have afforded more flexibility in their work hours and sick leave. Nonetheless, women across all occupational groups experienced some job and insurance-related difficulties following their diagnosis and treatment. However, participants who were overweight or obese, had incomes <$50,000 and full-time versus part-time workers tended to be the most vulnerable to employment problems. Prior research has found that overweight and obese women are more likely to experience employment problems, lower wages and discrimination than those of normal weight [25–27]. There is also a gender difference by weight in that women begin to see a decrease in wages at the overweight level, whereas men do not experience a decrease until they become obese [27]. Caliendo (2016) also found that “weight penalties,” including wage and employment problems, do not follow a linear relationship in women and that overweight and obese women in white-collar occupations tend to experience the worst wage penalties [28]. The results of this current study among breast cancer survivors are congruent with these previous findings.

One notable finding was that 35% of the young women in this study believed that they could not leave their current job, for fear of losing their health insurance. Reasons for this belief could have been related to concerns that their preexisting illness would preclude coverage by a new employer’s group health plan or their spouse’s/partner’s employer-based coverage. Some patients might have been able to be insured through a new health care plan, but at a much higher premium than prior to diagnosis. Both of these problems were common prior to the enactment of the Affordable Care Act (ACA) in 2010, which expanded health care coverage and lowered healthcare costs in the U.S. [22].

Insurance problems reported by the participants largely reflected an insured population. Participants experienced health insurance cost increases or being denied payment for a health care expense. Women adversely affected were those who were normal/underweight as compared to being obese and/or those with a tumor size > 5 cm as compared to < 2 cm. Both lower weight and a large tumor size are associated with higher stage disease, resulting in higher insurance costs and additional health concerns. In contrast, women taking adjuvant hormone therapy were less likely to report insurance problems. Being on hormonal therapy reflects the ER/PR status of the participants, with hormonal therapy being prescribed after the completion of initial cancer treatment to those who are ER+ and/or PR+, and has been proven to reduce the risk of a cancer recurrence or the development of a new breast cancer.

Since the data in this paper were collected, several federal laws now guarantee access to health insurance in the U.S., but do not regulate premiums [29, 30]. This may make it cost prohibitive for some cancer survivors to purchase individual health insurance if they do not qualify for group health insurance through an employer or other entity [21]. Studies conducted on the ACA and its implications for cancer survivors have shown promising findings. Cancer survivors currently have more insurance options through the ACA (via expanded Medicaid eligibility and premium tax credits) and have protection from being denied health insurance or paying much higher premiums due to pre-existing health conditions [31]. The passage of the ACA has eliminated denial of health insurance for a pre-existing condition in the U.S., but the affordability of health care through private health insurance companies can still make health insurance costs prohibitive for some young cancer survivors and their families.

Concerns about health care coverage can be a major factor in patients’ decisions to continue working during treatment or in their ability to reduce their work hours [3, 4]. Other patients who are able to leave their jobs or reduce their work hours during treatment, may have a difficult time finding new employment comparable to their old positions once their treatment has been completed or may not be offered to return to full-time work by a current employer [7]. All of these factors pose significant difficulties for younger cancer survivors.

What has not been discussed to a great extent in prior survivorship studies is the issue of life insurance. In this study we found that 11% of survivors had been denied life insurance following their breast cancer diagnosis. Life insurance protections do not always extend to patients with pre-existing conditions, whether they are applying for a new policy or trying to be added on a spouse’s/partner’s policy [32]. Life insurance might be available for some survivors, but the premiums may be unaffordable. These issues can be problematic for young patients, who often have younger children for whom to provide in the event of a poorer prognosis. This can add to the stress and anxiety experienced by survivors and their families.

Given the far reaching impact of breast cancer, health care professionals can assist breast cancer survivors in their work-related decisions through advice support and employer education [33]. Having tailored patient support and personal recommendations for work hours or circumstances, in addition to work-related adjustments, can increase the likelihood of cancer survivors remaining employed during their treatment, if necessary, or after their treatment [34]. A pilot study in The Netherlands created a 10-step plan for return to work, which aimed to improve communication between physicians in different departments and cancer survivors [35]. The plan especially focuses on working with occupational health professionals. Examples of advice included: “make sure the return-to-work plan encompasses the date and numbers of hours of the start” and “draw up a second, less ambitious return-to-work plan that may be used if the first plan fails.” Only 15% of patients in this pilot study created a second return-to-work plan, yet most considered a return date and wrote their first return-to-work plan.

Feuerstein et al. developed a cancer and work framework that takes into consideration the many factors affecting cancer survivors, including their health and well-being, functioning (physical, cognitive, emotional, interpersonal), and work demands [36]. It is aimed at conceptualizing the barriers to optimal work outcomes in cancer survivors and can be addressed by health care providers, survivors, and employers. Multidisciplinary models and guidelines, which connect physicians, nurse oncologists, and employers, help transition patients from cancer treatment to their intended employment status. Models such as these are useful in helping patients, their families and employers think through work-related issues both during and following cancer treatment. In general, breast cancer survivors have been found to have the greatest chance of returning to work compared to other cancers [17], and these models can assist survivors in maximizing their employment and work conditions following treatment.

There are several limitations to this study that should be noted. First, the study population was very homogenous with the majority identifying themselves as non-Hispanic white, married and well-educated. These factors could have potentially insulated the participants from problems related to their employment and insurance. A highly educated population is presumably better equipped to navigate insurance applications, policies, and reimbursement guidelines, and is better able to make adjustments in their work schedules. However, what was notable was that even among these study participants, between 27 and 40% reported job and/or insurance problems, suggesting that these problems are not confined to a single segment of breast cancer survivors.

A second limitation of this study is that the insurance and work-related issues identified by the participants were self-reported and were not verified through insurance or employer records. Additionally, insurance policies in the United States function differently than in Europe and other countries outside of North America. This may limit the generalizability of this study in terms of comparing it to countries with national health insurance. However, previous studies have shown that young breast cancer survivors in other countries are also vulnerable to losing work and social benefits [6]. It has been postulated that these concerns may therefore exceed the effects of specific insurance types [6].

Lastly, these data were collected prior to the enactment of the Affordable Care Act (ACA) in the United States [30]. However, given the participants’ occupations and higher educational status, the majority of the patients were insured and may not have had their insurance coverage affected as much as others by the enactment of the ACA. Thus, the findings of this study still have relevance to the insurance and employment issues that breast cancer patients face today. While certain aspects of the ACA are popular among almost all Americans (coverage for those with pre-existing conditions, coverage of dependents until age 26), certain provisions of the ACA are under review for modification or elimination. The uncertainty of provisions of this health care law may create more difficulties among young cancer survivors.

Strengths of the study include a large number of breast cancer survivors with available demographic and clinical data from medical chart review from which to examine correlates of employment and insurance issues. Additionally, 85% (708/836) of the original sample responded to the insurance and employment related questions. The majority of the patients had completed active therapy, and were approximately 18 months post-diagnosis. This timing makes the topic of return to work especially relevant since participants were at a time in their survivorship when they were returning to regular work hours and other aspects of their daily lives.

## Conclusions

In summary, these study results indicated that these young survivors experienced significant employment and insurance-related issues following their diagnosis and treatment for breast cancer. Given the high proportion of work years ahead for these women, insurance- and work-related issues should try to be anticipated and addressed prior to treatment to inform work expectations and avoid unnecessary insurance difficulties. Health professionals can assist in these efforts through advice and education about the treatment and recovery process.

## Data Availability

The datasets generated during the current study are not publicly available. This study was initiated and completed prior to the U.S. National Institutes of Health (NIH) and the U.S. Army Medical Research and Materiel Command data sharing guidelines. Data sharing may be available from the corresponding author on reasonable request.

## Abbreviations

ACA: Affordable Care Act
HMO: Health Maintenance Organization
